# HIV associated high-risk HPV infection among Nigerian women

**DOI:** 10.1186/1471-2334-13-521

**Published:** 2013-11-05

**Authors:** Sally N Akarolo-Anthony, Maryam Al-Mujtaba, Ayotunde O Famooto, Eileen O Dareng, Olayinka B Olaniyan, Richard Offiong, Cosette M Wheeler, Clement A Adebamowo

**Affiliations:** 1 Department of Nutrition, Harvard School of Public Health, 677 Huntington Avenue, Boston, MA 02115, USA; 2 Office of Strategic Information and Research Department, Institute of Human Virology Nigeria, 252 Herbert Macaulay Way, Abuja, Nigeria; 3 Department of Obstetrics and Gynecology, National Hospital, Abuja, Nigeria; 4 University of Abuja Teaching Hospital, Gwagwalada, Abuja, Nigeria; 5 Department of Pathology, University of New Mexico Health Sciences Center, Albuquerque, NM 87131, USA; 6 Institute of Human Virology and Greenebaum Cancer Center, University of Maryland School of Medicine, Baltimore, MD 21201, USA

**Keywords:** HIV, HPV, Nigeria

## Abstract

**Background:**

In developed countries, the incidence of cervical cancer has remained stable in HIV+ women but the prevalence and multiplicity of high-risk HPV (hrHPV) infection, a necessary cause of cervical cancer, appears different comparing HIV+ to HIV- women. Little is known about HIV and HPV co-infection in Africa.

**Methods:**

We enrolled women presenting at our cervical cancer screening program in Abuja, Nigeria between April and August 2012, and collected information on demographic characteristics, risk factors of HPV infection and samples of exfoliated cervical cells. We used Roche Linear Array HPV Genotyping Test® to characterize prevalent HPV and logistic regression models to estimate the association between HIV and the risk of hrHPV infection.

**Results:**

There were 278 participants, 54% (151) were HIV+, 40% (111) were HIV-, and 6% (16) had unknown HIV status. Of these, data from 149 HIV+ and 108 HIV- women were available for analysis. The mean ages (±SD) were 37.6 (±7.7) years for HIV+ and 36.6 (±7.9) years for HIV- women (*p*-value = 0.34). Among the HIV+ women, HPV35 (8.7%) and HPV56 (7.4%) were the most prevalent hrHPV, while HPV52 and HPV68 (2.8%, each) were the most prevalent hrHPV types among HIV- women. The multivariate prevalence ratio for any hrHPV and multiple hrHPV infections were 4.18 (95% CI 2.05 – 8.49, *p*-value <0.0001) and 6.6 (95% CI 1.49 – 29.64, *p*-value 0.01) respectively, comparing HIV + to HIV- women, adjusted for age, and educational level.

**Conclusions:**

HIV infection was associated with increased risk of any HPV, hrHPV and multiple HPV infections. Oncogenic HPV types 35, 52, 56 and 68 may be more important risk factors for cervical pre-cancer and cancer among women in Africa. Polyvalent hrHPV vaccines meant for African populations should protect against other hrHPV types, in addition to 16 and 18.

## Background

Human papillomavirus (HPV) is the most common sexually transmitted infection and at least 50% of sexually active people will get HPV at some time in their lives[1,2]. More than 100 HPV genotypes have been identified based on the sequence of their L1 genes [1,3]. HPV are classified into high-risk, probable high-risk and low-risk types, based on HPV-type-specific odds ratios and HPV prevalence among groups of women with cervical cancer and their controls [4]. HPV types 16, 18, 31, 33, 35, 39, 45, 51, 52, 56, 58, 59, 68, 73, and 82 are considered high-risk HPV (hrHPV) [4,5]. In addition to already established types, the International Agency for Cancer Research (IARC) recently classified HPV39, 59, 51 and 56 as carcinogenic while HPV68, 26, 30, 34, 53, 66, 67, 69, 70, 73, 82 and 85 were classified as possibly carcinogenic [6,7], but this classification has been criticized for lack of supporting epidemiological data [8]. The classification of HPV types according to their oncogenic potential is an ongoing process and is dependent on availability of data from different parts of the world.

Persistent hrHPV infection is recognized as a necessary but not sufficient cause for Cervical Intraepithelial Neoplasm (CIN) grades 2/3 and cervical cancer [9]. Molecular genetic studies of hrHPV from most parts of the world suggest that types 16 and 18 are the most prevalent types associated with CIN2 + [10,11]. This has been supported by several meta-analyses [8,12]. However these studies contained few high quality data from Africa and their results are liable to be biased by the availability and source of data. In the latest meta-analysis for example, inclusion of data from Eastern Asia inflated the prevalence of HPV58 suggesting that similar situation may occur as more data accrue from other parts of the world [9,10,13-17].

Although the limited data available suggests that the incidence and mortality from cervical cancer in Africa has not changed significantly in the last few decades despite the HIV/AIDS epidemic [18-21], HIV infection may result in different HPV distributions in cancer, within sub-Saharan Africa [22]. Several studies show that HIV+ women are more likely to be infected with non-16 and non-18 hrHPV types including HPV51, 53 and 56 as well as with multiple infections [23,24]. Studies done in Africa to date suggest that the most prevalent hrHPV types are HPV16, 52, 53 and 58 in HIV+ women, compared to HPV52 and 51 in HIV- women [16,25,26]. However, these studies were based on East African populations, in Uganda, Rwanda and Zambia. There is scarce data about the prevalent hrHPV among HIV+ women in West African populations. One study found the most prevalent types of hrHPV among women in Abidjan, Cote d’Ivoire to be HPV16 and 35, regardless of HIV status [27]. Given that cervical cancer incidence is ~50% higher in East compared to West Africa [28,29] we hypothesized that the prevalence, types and multiplicity of hrHPV infections might differ between East and West African populations and are likely to be associated with the rates of cervical cancer in these populations.

## Methods

### Study population

Between April and August 2012, we enrolled 278 women from 3 cervical cancer screening clinics at National Hospital Abuja and University of Abuja Teaching Hospital, Nigeria. All the study participants were 18 years or older, had prior vaginal sexual intercourse, not pregnant and had an intact uterus. Interviewer administered questionnaires were used to collect data on socio-demographic characteristics, HIV status, sexual and reproductive history. Participants HIV status were confirmed from their medical records. Trained nurses performed pelvic examinations on all the study participants. Samples of exfoliated cervical cells were obtained from the cervical os using Ayres spatula and stored at −80°C, until processing for HPV genotyping.

### HPV detection by genotyping

HPV DNA genotyping was done using linear array to HPV genotyping test (Roche Diagnostics), a qualitative in vitro test which utilizes amplification of target DNA by the Polymerase Chain Reaction (PCR) and nucleic acid hybridization and detects 37 high- and low-risk HPV genotypes [30]. The Linear Array HPV genotyping test has been validated and offers reliable and sensitive approach for detecting HPV DNA in cervical specimens, using standardized quality-controlled reagents [31-35].

### Data management

Data was managed using REDCap electronic data capture tools, hosted at the Institute of Human Virology, Nigeria (IHVN) [36,37].

### Statistical analysis

A total of 278 participants were enrolled in this study. We excluded 16 persons whose HIV status were not confirmed and 5 persons who had missing data on HPV genotype or several demographic variables. Descriptive analyses were performed to characterize the sampled population. *t*-tests were used to assess differences in the distribution of continuous variables between groups, while *χ*^2^ and Fishers exact tests were used for categorical variables. Multivariate logistic regression models were conducted to examine the association between HIV status and risk of hrHPV infections. All analyses were performed using SAS 9.3 for UNIX statistical software (SAS Institute, Cary, NC, USA).

### Ethics

The study was conducted according to the Nigerian National Code for Health Research Ethics and the Declaration of Helsinki. Ethical approval to conduct this study was obtained from the IHVN health research ethics committee. Informed consent was obtained from all participants before enrollment in the study.

## Results

We analyzed data on 257 women, of whom 58% (149/257) were HIV+ and 42% (108/257) were HIV-. The mean age and standard deviation (±SD) of the participants was 36.6 (±7.9) years for the HIV+ and 37.6 (±7.7) years for the HIV- women. Half (51%) of the HIV+ women were married, compared to 76% of the HIV- women who were married. The mean age at sexual initiation was 20.8 (±4.4) years for the HIV+ women, compared to 19.2 (±3.9) for the HIV- women, *p-value* = 0.004. HIV+ and HIV- women did not differ by total number of sexual partners or consistent condom use. Table 1 describes the demographic characteristics of the study participants by HIV status.

**Table 1:** Characteristics of the study population, by HIV status

**Characteristics**	**HIV positive (n = 149)**	**HIV negative (n = 108)**	**HIV positive (n = 149)**	**HIV negative (n = 108)**	**p-value**
	**Mean (±SD)**	**Mean (±SD)**	**N (%)**	**N (%)**	
**Age**^**#**^	36.6 (±7.9)	37.6 (±7.7)			0.34
**Age categories**^**#**^					0.30
- ≤30			35 (23.7)	23 (21.3)	
- 31 – 36			44 (29.7)	22 (20.4)	
- 37 – 44			47 (31.7)	44 (40.7)	
- ≥ 45			22 (14.9)	19 (17.6)	
**Age at sexual initiaton**^**#**^	20.8 (±4.4)	19.2 (±3.9)			0.004
**Age at sexual initiation**^**#**^					0.005
- < 18			41 (28.7)	21 (19.4)	
- 18 – 21			68 (47.5)	50 (46.3)	
- 22 - 25			26 (18.2)	16 (14.8)	
- > 25			8 (5.6)	21 (19.5)	
**Total sex partners**	3.3 (±3.2)	3.9 (±5.5)			0.34
**Total sex partners**					0.54
- 1			35 (23.7)	34 (31.5)	
- 2 – 3			68 (46.0)	40 (37.0)	
- 4 – 5			27 (18.2)	18 (16.7)	
- 5+			18 (8.1)	15 (13.8)	
**Education**					0.002
- ≤ 6 years			28 (18.8)	7 (6.5)	
- Secondary			104 (69.8)	76 (70.4)	
- Tertiary			17 (11.4)	25 (23.1)	
**Marital Status**					<0.001
- Married, %			76 (51.0)	82 (75.9)	
- Not married, %			73 (49.0)	26 (24.1)	
Condom use*					0.26
- Yes			3 (2.0)	0 (0)	
- No			146 (98.0)	108 (100)	

^#^Age in years.

*Consistent condom use in the past 2 years.

**Figure 1:**
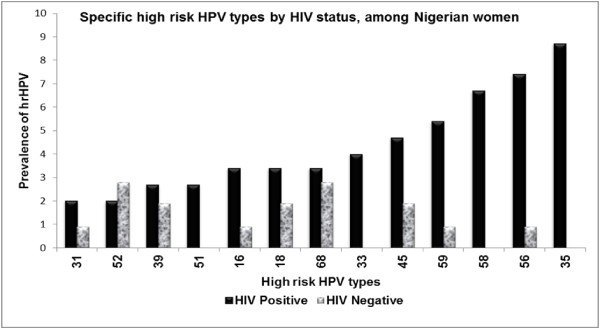
Frequency of high risk HPV types among Nigerian women, by HIV status.

The prevalence of hrHPV infection was 25% (64/257). Among the HIV+ women, 36% (53/149) had hrHPV infections, while 10% (11/108) of the HIV- women had hrHPV infections (*p* = <0.001). Thirteen specific hrHPV types were detected among the HIV+ women and 9 hrHPV types among the HIV- women. HPV33, 35, 51 and 58 were detected only among the HIV+ women. The most prevalent hrHPV type in the study population was HPV35; it was detected exclusively in HIV+ women, where it accounted for 24.5% (13/53) of the hrHPV infections. HPV33, 51 and 58 were also detected only in the HIV+ women. The prevalence of HPV types 35, 56 and 58 were significantly different among HIV+ compared with HIV- women (Table 2). Figure 1 shows the specific hrHPV types among the women, by HIV status

**Table 2:** Prevalence of specific high-risk HPV types by HIV status, among Nigerian women

**HrHPV type**	**HIV positive [n = 149]**	**HIV negative [n = 108]**	**p-value**
	**N (%)**	**N (%)**	
16	5 (3.4)	1 (0.9)	0.41
18	5 (3.4)	2 (1.9)	0.70
31	3 (2.0)	1 (0.9)	0.64
33	6 (4.0)	0 (0.0)	0.04
35	13 (8.7)	0 (0.0)	<0.001
39	4 (2.7)	2 (1.9)	1.0
45	7 (4.7)	2 (1.9)	0.31
51	4 (2.7)	0 (0.0)	0.14
52	3 (2.0)	3 (2.8)	0.69
56	11 (7.4)	1 (0.9)	0.01
58	10 (6.7)	0 (0.0)	0.006
59	8 (5.4)	1 (0.9)	0.08
68	5 (3.4)	3 (2.8)	1.0

We found single hrHPV infections were more common 66% (42/64), compared to multiple hrHPV infections 34% (22/64) in the overall study population (*p* = <0.001). Among the HIV+ women, 14% (21/149) had multiple hrHPV infections while 2% (2/108) of the HIV- women had multiple hrHPV infections (*p* = <0.001). Of the 21 HIV+ women infected with multiple hrHPV genotypes, 15 were infected with two, 5 were infected with three and 1 was infected with six hrHPV types. All the HIV+ women infected with 2 hrHPV types had HPV35 and another type.

The age adjusted prevalence ratio (PR) of any instance of single hrHPV infection and any instance of multiple hrHPV infection comparing HIV+ women to HIV- women were 4.71 (95% CI 2.34 – 9.46, *p* = <0.001) and 8.68 (95% CI 1.99 – 37.96, *p* = <0.004) respectively. The multivariate PR of any hrHPV and multiple hrHPV infection adjusted for age and education were 4.18 (2.06 - 8.49 *p* = <0.001) and 6.66 (1.50 - 29.64 *p* = 0.01) respectively. Education accounted for most of the variation in these multivariate models (Table 3).

**Table 3:** Prevalence ratios and 95% confidence intervals of high-risk HPV infections, among Nigerian women

		**Single high risk HPV**		**Multiple high risk HPV**	
		**PR (95% CI)**	***p*-value**	**PR (95% CI)**	***p*-value**
**Model 1**	HIV (Ref = HIV negative)	4.18 (2.06 - 8.49)	<0.001	6.66 (1.50 - 29.64)	0.01
	Age	0.94 (0.90 - 0.98)	0.001	0.95 (0.89 - 1.01)	0.10
	Education	0.58 (0.32 - 1.04)	0.06	0.29 (0.12 - 0.70)	0.006
**Model 2**	HIV (Ref = HIV negative)	4.76 (2.28 - 9.93)	<0.001	7.97 (1.80 - 35.29)	0.006
	Age	0.93 (0.89 - 0.97)	0.001	0.95 (0.89 - 1.01)	0.08
	No. of sexual partners	0.91 (0.65 - 1.28)	0.58	0.80 (0.48 - 1.34)	0.40
	Age at sexual initiation	0.94 (0.66 - 1.35)	0.74	0.71 (0.40 - 1.26)	0.23
**Model 3**	HIV (Ref = HIV negative)	4.38 (2.06 - 9.30)	0.001	5.93 (1.29 - 27.14)	0.02
	Age	0.93 (0.89 - 0.97)	0.001	0.95 (0.89 - 1.01)	0.11
	Education	0.64 (0.34 - 1.21)	0.17	0.31 (0.12 - 0.81)	0.02
	Marital status	1.03 (0.53 - 2.01)	0.93	1.82 (0.68 - 4.87)	0.23
	No. of sexual partners	0.93 (0.65 - 1.32)	0.68	0.78 (0.45 - 1.33)	0.36
	Age at sexual initiation	1.00 (0.69 - 1.45)	0.99	0.91 (0.50 - 1.64)	0.74

## Discussion and conclusion

Our study shows that the prevalence of hrHPV infection was significantly higher among HIV+ women, compared to HIV- women and HIV+ women were more likely to have multiple hrHPV infections. In decreasing order, HPV35, 56, 58, 59 and 45 were the most prevalent types of hrHPV infection found among those who were HIV+ while HPV68, 52, 39, 45 and 18 were the most prevalent types of hrHPV infection among those who were HIV-, in our study participants.

Our results are consistent with the findings from other studies that showed women with HIV infection were more likely to be infected with non-16 and non-18 hrHPV types [24,38,39]. The most prevalent types of hrHPV found in this study differ from those in other populations. Among 208 HIV+ non-pregnant women in São Paulo, Brazil and 229 HIV+ non-pregnant women in New York, US, the most common types of hrHPV were 51, 18, 16 and 56, 53, 16, 58 respectively [23,24]. Among HIV+ pregnant women in Thailand, the most common hrHPV types were 39, 52, 53 and 16 [39]. In Africa, a study among HIV+ and HIV- women in Kampala, Uganda found the most prevalent types of hrHPV to be 52, 16 and 58, and these where similar to findings in Nairobi, Kenya [25,40]. In Kigali, Rwanda, the most prevalent hrHPV type among HIV+ women was also HPV52, followed by HPV51 and 58 [16].

Our findings were similar to the results from other studies in Nigeria. Cage et al. found non-16 and non-18 HPV were the most prevalent hrHPV types [41]; Okolo et al. found HPV35 was as prevalent as HPV16; Musa et al. found the prevalence of hrHPV among HIV+ women was 45% [42]. Lifestyle factors such as socio-cultural characteristics, nutritional, environmental, sexual behavior and hygiene, vaginal microbiota and genetic factors, along with specific geographic distribution of hrHPV types may explain the varying prevalence of hrHPV and cervical cancer incidence across populations. The incidence (52.8 per 100,000) of cervical cancer in Zambia, East Africa is ~50% higher than in Nigeria, West Africa where the incidence is 34.5 per 100,000 [18,28,29]. The differences in the prevalent types of hrHPV in West and East Africa, may partly explain these regional variations in cervical cancer incidence.

Other studies, like ours, found a high prevalence of multiple hrHPV infections among HIV+ women [16,24-27,38,39]. The full spectrum of hrHPV types that are involved in multiple infections, their persistence, individual and relative contribution to oncogenicity and duration of persistence of the different hrHPV types in the context of multiple infections in African women is not known. The etiological and preventative significance of multiple infections and its potential impact on current vaccination and HPV DNA based testing strategies are also not entirely clear and should be assessed in different populations [16,17].

Differences in epidemiology of hrHPV between developed and developing countries may be meaningful, given that the current and next generations of HPV vaccines do not include some of the types that are prevalent in Africa. This will be particularly significant if there is little or no antibody cross-reactivity between current vaccines and the hrHPV types prevalent in Africa [8]. Given the high prevalence of non-16 and non-18 hrHPV among HIV+ women in Nigeria and other African countries, current vaccines may have limited impact in this section of the population. Longitudinal investigations of HPV genotype-specific risks for cervical precancer and cancer outcomes should be conducted in Africa. As the incidence of cervical cancer among HIV+ women has increased in the combination Antiretroviral Therapy era, there is a need for further studies examining the role of covariates of persistent hrHPV infection such as sexual behavior, sexual hygiene, diet, smoking, alcohol consumption, concurrent genital tract infections, other illnesses and sexual partner health behavior in cervical carcinogenesis.

## Abbreviations

CI: Confidence interval; CIN: Cervical intraepithelial neoplasm; HIV+: HIV positive; HIV: HIV negative; HPV: Human papillomavirus; hrHPV: high-risk HPV; IARC: International Agency for Cancer Research; IHVN: Institute of Human Virology Nigeria; PCR: Polymerase chain reaction; PR: Prevalence ratio.

## Competing interests

CMW has received through the University of New Mexico, funds from grants and cooperative agreements from the US National institutes of Health related to cervical screening, funds from GSK for HPV vaccine studies and reimbursements for travel related to publication activities and equipment and reagents from Roche Molecular Systems for HPV genotyping. Other authors report no conflicts of interest.

## Authors’ contributions

SNA analyzed the data and drafted the manuscript. MA, AOF, and EOD contributed to the study coordination. OO and RU, site investigators, contributed to the study design, implementation and provided revisions of the manuscript. CMW performed HPV genotyping, contributed to data interpretation and provided revisions of the manuscript. CAA conceived the study, obtained funds, contributed to the study design and provided critical revisions of the manuscript. All authors read and approved the final manuscript.

## Pre-publication history

The pre-publication history for this paper can be accessed here:

http://www.biomedcentral.com/1471-2334/13/521/prepub

## Study design

Cross-sectional survey.
